# Cryo-Biopsy versus 19G needle versus 22G needle with EBUS-TBNA endoscopy

**DOI:** 10.7150/jca.75589

**Published:** 2022-08-08

**Authors:** Rena Oikonomidou, Dimitris Petridis, Christoforos Kosmidis, Konstantinos Sapalidis, Wolfgang Hohenforst-Schmidt, Vagelis Christakidis, Savas Petanidis, Dimitris Mathaios, Eleni Isidora Perdikouri, Sofia Baka, Christos Tolis, Anastasios Vagionas, Bojan Zaric, Aris Ioannidis, Marios Anemoulis, Konstantinos Porpodis, Vasileios Papadopoulos, Paul Zarogoulidis

**Affiliations:** 1 Health Center of Evosmos, Thessaloniki, Greece.; 2 3rd Department of Surgery, “AHEPA” University Hospital, Aristotle University of Thessaloniki, Medical School, Thessaloniki, Greece.; 3 Department of Food Technology, School of Food Technology and Nutrition, Alexander Technological Educational Institute, Thessaloniki, Greece.; 4 Sana Clinic Group Franken, Department of Cardiology / Pulmonology / Intensive Care / Nephrology, ''Hof'' Clinics, University of Erlangen, Hof, Germany.; 5 Oncology Department, General Hospital of Serres, Serres, Greece.; 6 Department of Pulmonology, I.M. Sechenov First Moscow State Medical University, Moscow, Russian Federation.; 7 Oncology Department, General Hospital of Rhodes, Rhodes, Greece.; 8 Oncology Department, General Hospital of Volos, Greece.; 9 Oncology Department, “Interbalkan” European Medical Center, Thessaloniki, Greece.; 10 Oncoderm, Private Oncology Clinic, Ioannina, Greece.; 11 Oncology Department, General Hospital of Kavala, Kavala, Greece.; 12 Faculty of Medicine, University of Novi Sad, Institute for Pulmonary Diseases of Vojvodina, Novi Sad, Serbia.; 13 Surgery Department, Genesis Private Hospital, Thessaloniki, Greece.; 14 General Surgery Department, General Clinic Euromedica, Thessaloniki, Greece.; 15 Pulmonary Department, “G. Papanikolaou” General Hospital, Aristotle University of Thessaloniki, Thessaloniki, Greece.; 16 Oncology Department, University General Hospital of Larissa, University of Thessali, Larissa, Greece.; 17 Pulmonary-Oncology Department, “General Clinic” Private Clinic, Thessaloniki, Greece.

**Keywords:** radial-ebus, Linear-EBUS, 22G needle, 21G needle, 19G needle, cryo-biopsy, bronchoscopy, lung cancer, cancer

## Abstract

**Introduction:** We have been using cryo-biopsy for endobronchial lesions for lung cancer diagnosis and debulking. Cryo-biopsy is also known to be an excellent tool for diagnosis of lung interstitial disease. Recently cryo-biopsy with the 1.1mm probe was used for lymphnode biopsy.

**Patients and Methods:** 311 patients participated with lymphadenopathy and at least one lung lesion. The following tools were used for diagnosis; 22G Mediglobe Sonotip, 22G Medigolbe, 21G Olympus, 19G Olympus and 1.1mm cryo probe ERBE CRYO 2 system (3 seconds froze). A PENTAX Convex-probe EBUS was used for biopsy guidance.

**Results:** Cell-blocks slices had a higher number in the 19G needle group (19G> Cryo Probe>22G Mediglobe Sonotip >21G Olympus >22G Mediglobe).

**Conclusion:** Cryo biopsy of the lymphnodes is safe with the 1.1mm cryo probe. Further studies are needed in order to evaluate new probes and the technique specifications.

## Introduction

Lung cancer is still diagnosed at advanced stage due to early disease symptoms. In the past five years all efforts are directed to early disease diagnosis by performing low-dose computed tomography scans with intravenous contrast administration in high risk patients for lung cancer 1. We can use the radial endobronchial ultrasound or an electromagnetic navigation system for diagnosis of pulmonary nodules or lung cancer lesions 2, 3. We can also use the rapid on site technique (ROSE) in order to assess our sample as a method of fast diagnosis and evaluation of our sample 4. Convex probe endobronchial ultrasound will then provide us with the staging of the mediastinum with a 22G or a 21G needle 5. The sample size of 22G needle and 21G needle is efficient for the diagnosis of lung cancer and metastatic cancers 6. The sample and the current techniques allow the molecular investigation of this small size sample with next generation sequencing (NGS) 7, 8. Positron emission tomography-Computed tomography (PET-CT) will complete the staging 9. In the recent five years cryo biopsies have been extensively used for the diagnosis of diffuse lung disease due to the larger tissue sample 10. This was made possible with the use and experience of radial-endobronchial ultrasound and the new electromagnetic systems which were used for guidance of the cryo-probe 11-13. In the case of endobronchial lung cancer cryo biopsies have been extensively used 14. However; in the case of lymphadenopathy until now we have been using 22G, 21G and 19G needles as a minimal invasive technique compared to mediastinoscopy 15. An effort has been made to acquire more sample and therefore the cryo probe 1.1mm has been used with an ERBE CRYO 2 system 16, 17. There are no technique specifications and there are ongoing studies in the field, we performed this study in order to enlighten the sample size.

## Patients and Methods

### Patients

Three hundred and eleven patients ≥18 years of age with lymphadenopathy and at least one lung lesion were recruited. Our investigational review board of Aristotle University of Thessaloniki approved the protocol. We performed the procedure for diagnostic reasons and in order to evaluate the sample size of different needles and methods with the method of cell-block slices. The patients had to be fit for sedation and all of them had lymphnodes from 1cm up to 3cm centimeters. Ninety three were diagnosed either with lymphoma or metastasis from another site.

### Methods

A PENTAX Convex-probe was used in order to take samples with 22G Mediglobe needle (Fine needle aspiration), 22G Mediglobe Sonotip needle (Fine needle aspiration), 21G Olympus needle (Fine needle aspiration), a 19G Olympus needle (Fine needle aspiration biopsy) and Cryo probe 1.1mm. In specific based on the following two publications Franke K-J. et. al. 17 and Gonuguntla H-K. et. al. 16 regarding the cryobiopsy procedure we performed EBUS-TBNA biopsy with a 19G needle and then we pushed the 1.1mm Cryo probe that was connected to an ERBE CRYO 2 system into the convex-probe working channel and inserted it in the previous biopsy site and activated the probe for 3seconds. We performed the procedure 2 times and we noticed that the bleeding was tolerable and in 5 cases we used special hemostatic aerosol by the company HYGEIASIS, Thessaloniki, Greece. Regarding the needle biopsies for each different needle model we performed 4 passes. Cell-blocks were created for every patient and slices were made in order to evaluate the sample size. The patients were under sedation with jet-ventilation and they were either intubated or had a laryngeal mask. Fourty nine patients had biopsy with 22G Mediglobe FNA needle, fifty four had biopsy with a 22G Mediglobe Sonotip Needle, fifty six had biopsy with a 21G Olympus needle, eighty nine with a 19G needle and sixty three with the Cryo probe.

### Statistical analysis

The number of slices was chosen as the key variable potentially affected by the action of the variables: sex, method of penetration, lesion size, cancer type and number of cell blocks.

Thus, the number of slices considered as the dependent variable was regressed against the aforesaid independent variables, and their important effect was determined by the forward selection of each variable checking at p <0.05 probability level each significant entrance in the model. Variables were then ranked according to the magnitude of their effects using the logworth value, that is -ln(p-value).

## Results

**Figure 1:**
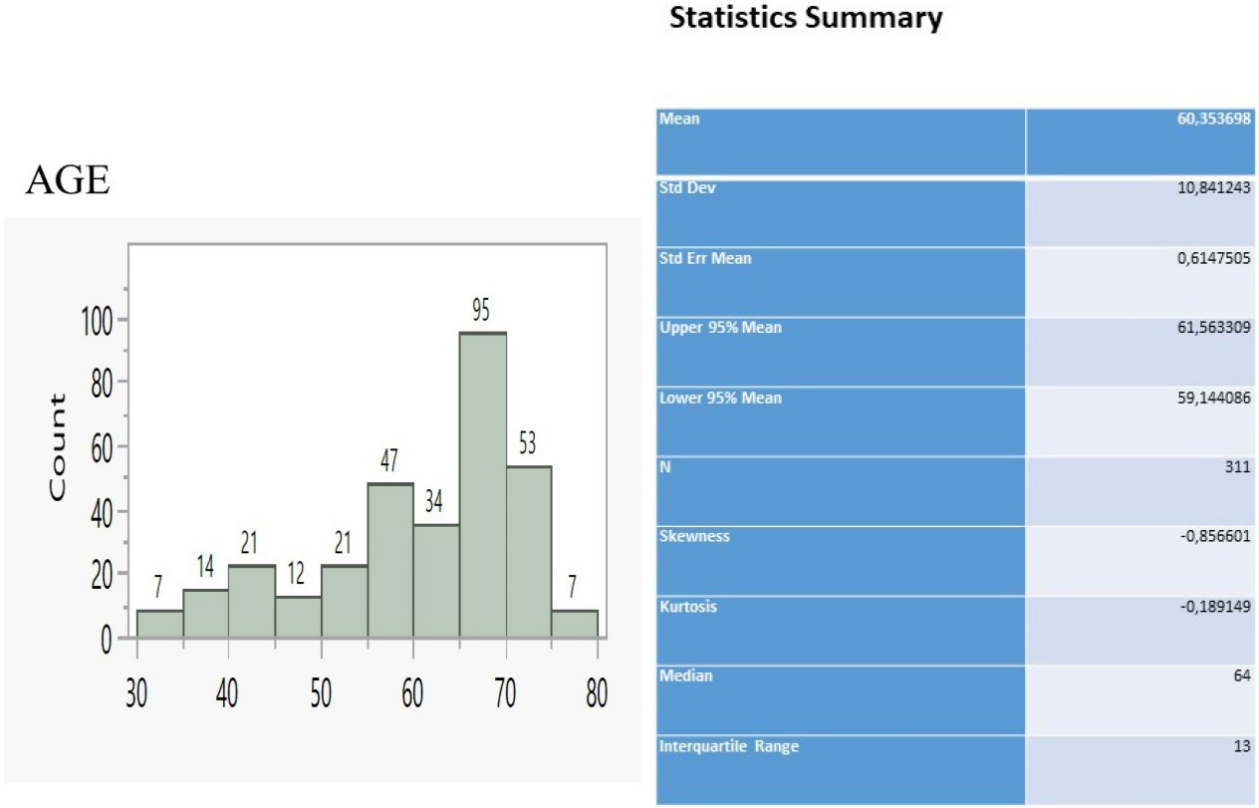
Age distribution and relevant statistics.

The sample of patients consisted of 311 individuals ranging between 30 and 80 y.o. (Figure 1) and having the main bulk to be distributed between 55 and 75 y.o (229 patients, 73.6%). We considered that 22G Mediglobe FNA needle and cryo probe are the “small” tip techniques and that the 22G Mediglobe Sonotip FNA, 21G Olympus FNA and 19G Olympus FNAB are the large tip techniques based the diameter of the tip in order to make the statistics easier.

Table 1 demonstrates the frequency tabulation of parameters categories, also deploying the mean numbers of slices per each variable category. Accordingly, men prevail over women at a ratio 1,4:1 (181:130) with similar mean numbers of slices (7.8 to 8.9). The five techniques of needle and probe penetration disclose the presence of two groups, one with small content (22G Mediglobe FNA and Cryoprobe) and 4,1 mean slices and another group of needles with large content and 11 mean slices (see Method 2, Table 1).

The size of lesions was divided into three increasing levels corresponding also to increased number of slices. One cell block was found in 163 cases (52.4%), but increasing numbers of slices were recorded only for 2 and 3 cell blocks. The Hodhgin cancer clearly distinguishes from the other types providing 14 mean slices instead of the others' common average of 8 slices. Finally, the tissue content in the needles facilitates a further increase of slices by 2 (7 to 9).

**Figure 2:**
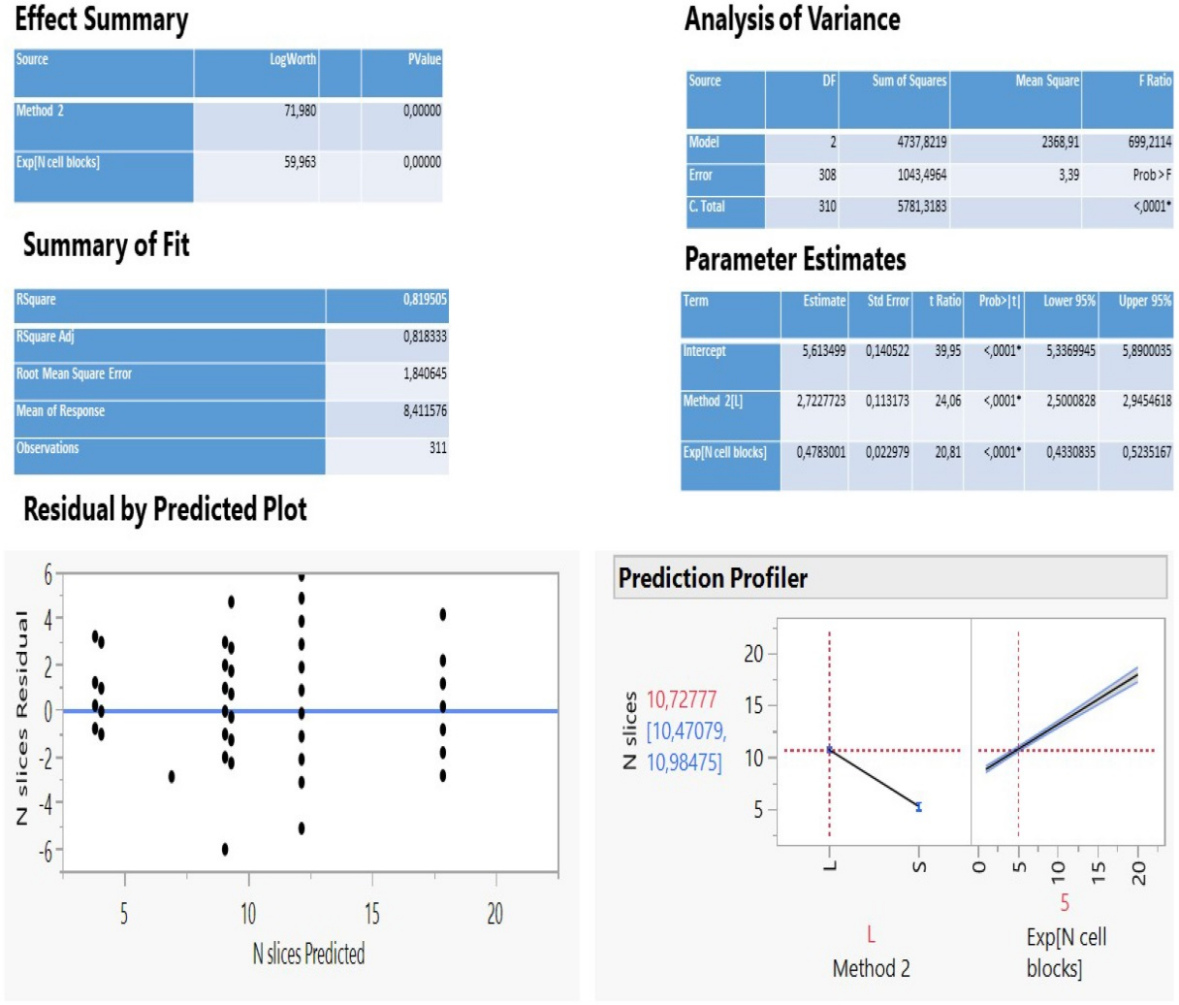
Statistical information of the multiple regression of N slices on method groups and N cell blocks (exponentially transformed).

The analysis of multiple regression of N slices against all other parameters statistically selected only two important variables (method 2 and N cell blocks exponentially transformed) to describe adequately a predictive model (Figure 2) as the two fit R^2^ coefficients (determined and adjusted, 81.2%) and the scattered distribution of residuals vs predicted values so indicated.

**Figure 3:**
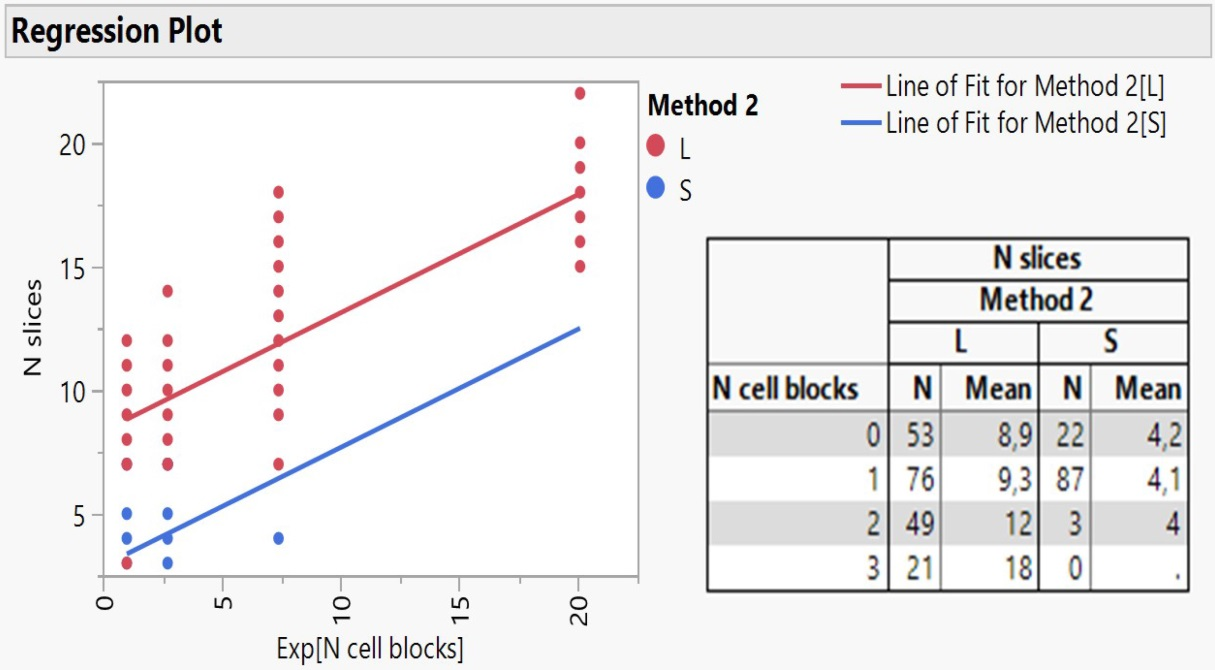
Regression plot of N slices on exponentially transformed N cell blocks as divided by the method groups accompanied by the relevant cross-tabulation.

The method 2 explains 56.6% of the total variation of the model (logworth 71.98) and the N cell blocks another 24.6% (logworth 59.96). The predicted plot in Figure 2 reveals a significant decrease of slices creation (5.3) using either the needle 22G Mediglobe FNA or the cryoprobe and a two-fold increase when operation carries out with any other needle (10.7 slices). Also, as the number of cell blocks increase, more slices are expected to be dissected according to the rate coefficient 0.478 on an exponential scale. This can be better explained using the natural scale as follows: no blocks (ln1) correspond initially to 8.3 slices, 1 block (ln 2.7) produces 9.6 slices, 2 blocks (ln7.4) create 11.9 slices and 3 blocks (ln20) 17.9 slices (Figure 3).

Taking a more insightful look in the regression lines as better exemplified by embedding the two groups of method 2 (S and L) and figure 3 shows, it appears that only the large needle content facilitates the creation of slices covering the full range of cell blocks, while the small content responds to only one block. Furthermore, the cross-tabulation of N slices per cell block and needle content reveals a very close agreement between predicted and actual values of mean slices, strongly enhancing the reliability of regression model attempted in the present study. Cryo biopsies were not superior in terms of sample volume, at least with our methodology. We present the following algorithm according to the larger volume to the lowest 19G Olympus > Cryo biopsy > 22G Mediglobe Sonotip > 21G Olympus > 22G Mediglobe.

## Discussion

**Figure 4:**
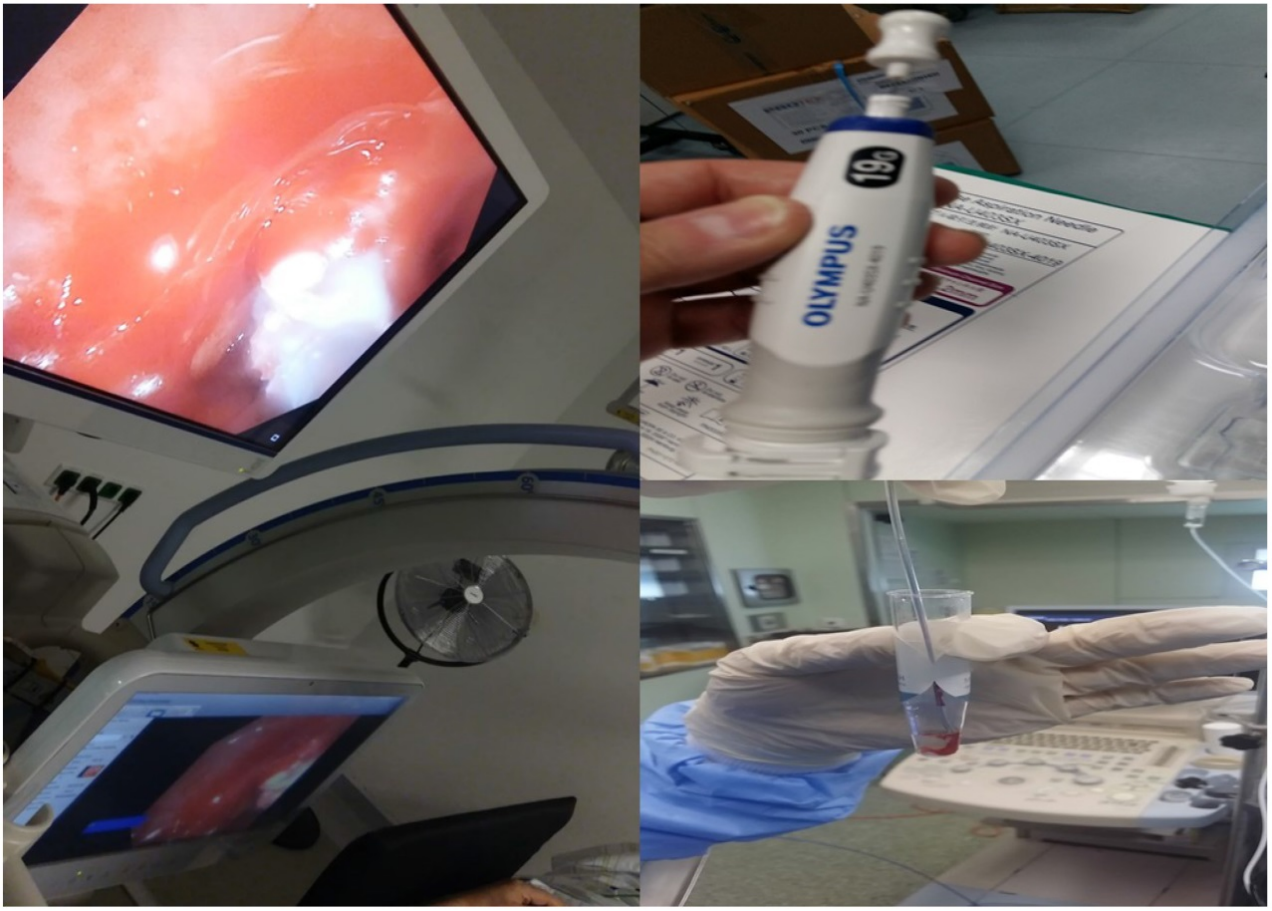
In specific (new Figure 4) on the left we can see a cryoprobe 1.1mm pulling out from a lesion a small tissue sample, while on the upper right we can see a 19G Needle and lower right the tissue sample released inside a cytolite plastic bottle.

It has been previously presented that indifferent of the needle size the sample is enough for diagnosis and molecular next generation sequencing (NGS) investigation for lung cancer and other metastatic cancers in the lung parenchyma 5, 15, 18. In a recent study it was observed that the 19G needle when compared to 22G needle has a larger sample size and it has a higher diagnostic sensitivity for lymphoma 19, 20. This information is in accordance with previous publications with the esophageal ultrasound (EUS) where the gastroenterologists used larger 19G needles 21, 22. However; based on the published literature regarding cryo biopsies for endobronchial lesions it was observed that larger samples were obtained 23. The new ERBE CRYO 2 system has a 1.1mm cryo probe which is small enough to go through a convex probe endoscope or even a 19G sheath for more accurate guidance (Figure 4).

Based on this technology advancement an effort has been made to acquire more sample by using cryo biopsies it remains to be clarified after several studies which is the most efficient methodology. An experiment was conducted in pigs and a cases series were recently published describing a different methodology 16, 17. Major limitation of our study was patients' selection and the non-homogeneity of the casuistic regarding lymph node size, type and region. Moreover; we used different sample methods in different patients creating bias in terms of EBUS-TBNA results. In our study an evaluation of different needles versus cryo biopsy was performed at least with our methodology (Table 2).

**Figure 5:**
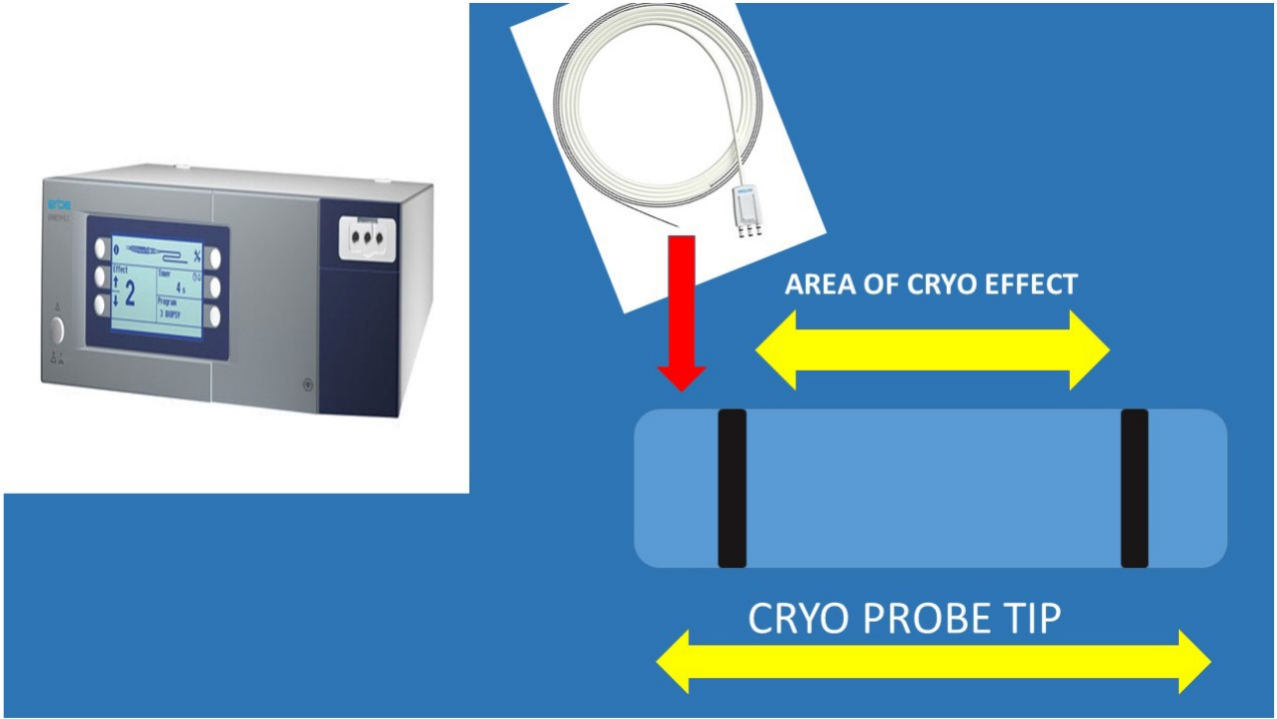
Cryo ERBE system with a new cryo probe tip proposal. As presented the inner area of the tip and only on the sideways will have the cryo effect in order not to destroy the barriers of the lymphnode. Also, the tip should be sharp and the cable probe stronger.

The cryo probe was not inserted through the 19G needle protection needle protection sheath, there was a way to take out the metallic part of the needle. It was observed that the cryo probe was too sensitive and was easily damaged. Therefore it was not cost-effective to continue by inserting the cryo probe through the sheath. In our previous published studies we established our methodology by evaluating the sample size by the number of slices created by a cell-block, this is a method to quantify the sample size 5. Another issue observed was the delay for the cryoprobe biopsy where for the 19G needle biopsies we needed up-to 30 minutes, while for the cryoprobe we needed from 30-45minutes and of course more hemorrhage was observed without any significant issues. The larger the lesions, the easier the procedure of tunneling for the cryoprobe. In any case lymphoma is another issue since we need larger volume of solid tissue structure in order to diagnose this entity especially with Hodgkin lymphoma. In the case of Non-Hodgkin 22G needles are efficient based on numerus studies and case reports 5, 24. In our study cryo biopsies were not superior in terms of sample volume, at least with our methodology. The following algorithm is proposed according to the larger volume to the lowest 19G Olympus > Cryo biopsy > 22G Mediglobe Sonotip > 21G Olympus > 22G Mediglobe. The 19G Olympus needle is 4cm at length and in the case of large lymphnodes it can penetrate deep enough and acquire long core tissue samples and it is visible during the puncture procedure, while the cryo probe although visible, there is no way to actually predict the sample that you are going to extract and the possible damage that is done. The cryo probe cannot be forced into the lymphnode, a whole has to be created and therefore minor hemostasis issues can be observed, also, it is more sensitive that the 19G needle. Probably as in the case of CellVizio where a new probe was created that can get through the 19G needle and evaluate the lesion, another type of cryo probe has to be created. We propose the following structure and effect (Figure 5).

## Conclusion

It is proposed that this method could become more efficient after some modifications have been made to the cryo probe. Future studies will enlighten the best technique. Probably we will need a needle with sharp tip and a stronger main body as the sheath of the 19G needle.

## Figures and Tables

**Table 1:** Descriptive statistics of the parameters under study and relevant code notification. The variable Method 2 divides Method into two groups, small needle content, S (categories 1 and 5) and large content, L (categories 2-4).

	N slices				
Sex	N	Mean	Std Dev	Min	Max
0	130	7,8	4,68	3	19
1	181	8,9	3,99	3	22
Method					
1	49	4	0,78	3	5
2	54	10	1,51	8	14
3	56	8,4	1,49	3	11
4	89	13	4,08	7	22
5	63	4,2	1,21	3	7
Size					
1	103	7,8	3,24	3	17
2	132	8,4	3,62	3	18
3	76	9,3	6,22	3	22
N cell blocks					
0	75	7,5	2,67	3	12
1	163	6,5	2,94	3	14
2	52	12	3,56	4	18
3	21	18	1,8	15	22
Cancer type					
0	162	8,2	4,13	3	22
1	56	8,7	4,14	3	18
2	40	7,7	4,69	3	18
3	39	7,7	4,69	3	18
4	14	13	4,68	9	20
Tissue					
0	92	6,9	2,72	3	12
1	219	9	4,7	3	22
Method 2					
L	199	11	3,47	3	22
S	112	4,1	1,04	3	7

**Table 2:** Adverse effects per method and percentage.

	19G NEEDLE	CRYOPROBE	
HEMORAGE	2PTS	17PTS	
VENTILATION ISSUES	10PTS	25PTS	
COST	520EURO	1040EURO	ONLY FOR BIOPSY NEEDLE AND CRYO PROBE
TIME	20-30MIN	30-40MIN	

## References

[1] Nanavaty P, Alvarez MS, Alberts WM (2014). Lung cancer screening: advantages, controversies, and applications. Cancer control: journal of the Moffitt Cancer Center.

[2] Huang Z, Huang H, Ning Y, Han J, Shen Y, Shi H (2019). Radial probe endobronchial ultrasound assisted conventional transbronchial needle aspiration in the diagnosis of solitary peribronchial pulmonary lesion located in the segmental bronchi. Journal of Cancer.

[3] Shaller BD, Gildea TR (2020). What is the value of electromagnetic navigation in lung cancer and to what extent does it require improvement?. Expert review of respiratory medicine.

[4] Simon M, Pop B, Toma IL, Vallasek AK, Simon I (2017). The use of EBUS-TBNA and ROSE in the diagnosis of lung cancer. Romanian journal of morphology and embryology = Revue roumaine de morphologie et embryologie.

[5] Zarogoulidis P, Petridis D, Sapalidis K, Tsakiridis K, Baka S, Vagionas A (2020). Lung cancer biopsies: Comparison between simple 22G, 22G upgraded and 21G needle for EBUS-TBNA. Journal of Cancer.

[6] Yarmus LB, Akulian J, Lechtzin N, Yasin F, Kamdar B, Ernst A (2013). Comparison of 21-gauge and 22-gauge aspiration needle in endobronchial ultrasound-guided transbronchial needle aspiration: results of the American College of Chest Physicians Quality Improvement Registry, Education, and Evaluation Registry. Chest.

[7] Tsoulos N, Papadopoulou E, Metaxa-Mariatou V, Tsaousis G, Efstathiadou C, Tounta G (2017). Tumor molecular profiling of NSCLC patients using next generation sequencing. Oncology reports.

[8] Oezkan F, Khan A, Zarogoulidis P, Hohenforst-Schmidt W, Theegarten D, Yasufuku K (2014). Efficient utilization of EBUS-TBNA samples for both diagnosis and molecular analyses. OncoTargets and therapy.

[9] Farsad M (2020). FDG PET/CT in the Staging of Lung Cancer. Current radiopharmaceuticals.

[10] Mikolasch TA, Garthwaite HS, Porter JC (2017). Update in diagnosis and management of interstitial lung disease. Clinical medicine.

[11] Herath S, Yap E (2018). Novel hybrid cryo-radial method: an emerging alternative to CT-guided biopsy in suspected lung cancer. A prospective case series and description of technique. Respirology case reports.

[12] Sun J, Mao X, Xie F, Han B, Chen H (2015). Electromagnetic navigation bronchoscopy guided injection of methylene blue combined with hookwire for preoperative localization of small pulmonary lesions in thoracoscopic surgery. Journal of thoracic disease.

[13] Chan JWY, Lau RWH, Chu CM, Ng CSH (2021). Expanding the scope of electromagnetic navigation bronchoscopy-guided transbronchial biopsy and ablation with mobile 3D C-arm Machine Cios Spin((R))-feasibility and challenges. Translational lung cancer research.

[14] Bergner A, Huber RM (2011). [Interventional bronchoscopy in lung cancer]. Der Internist.

[15] Zarogoulidis P, Hatzibougias D, Tsakiridis K, Matthaios D, Hohenforst-Schmidt W, Huang H (2021). Lymphadenopathy and granulomas: benignancy of malignancy and differential diagnosis with endobronchial ultrasound-transbronchial needle biopsy 19G needle fine-needle aspiration biopsy. Lung cancer management.

[16] Gonuguntla HK, Shah M, Gupta N, Agrawal S, Poletti V, Nacheli GC (2021). Endobronchial ultrasound-guided transbronchial cryo-nodal biopsy: a novel approach for mediastinal lymph node sampling. Respirology case reports.

[17] Franke KJ, Nilius G, Ruehle KH, Enderle MD, Linzenbold W, von Weyhern CH (2013). The cryo-needle: a new tool for histological biopsies. A feasibility study. Lung.

[18] Zarogoulidis P, Papadopoulos V, Maragouli E, Papatsibas G, Karapantzos I, Bai C (2018). Tumor heterogenicity: multiple needle biopsies from different lesion sites-key to successful targeted therapy and immunotherapy. Translational lung cancer research.

[19] Zarogoulidis P, Huang H, Hu Z, Wu N, Wang J, Petridis D (2021). Priority of PET-CT vs CT Thorax for EBUS-TBNA 22G vs 19G: Mesothorax Lymphadenopathy. Journal of Cancer.

[20] Manley CJ, Kumar R, Gong Y, Huang M, Wei SS, Nagarathinam R (2022). Prospective randomized trial to compare the safety, diagnostic yield and utility of 22-gauge and 19-gauge endobronchial ultrasound transbronchial needle aspirates and processing technique by cytology and histopathology. Journal of the American Society of Cytopathology.

[21] Gines A, Fusaroli P, Sendino O, Seicean A, Gimeno-Garcia AZ, Gratacos-Gines J (2021). Performance of a new flexible 19 G EUS needle in pancreatic solid lesions located in the head and uncinate process: A prospective multicenter study. Endoscopy international open.

[22] de Nucci G, Petrone MC, Imperatore N, Forti E, Grassia R, Giovanelli S (2021). Feasibility and Accuracy of Transduodenal Endoscopic Ultrasound-Guided Fine-Needle Aspiration of Solid Lesions Using a 19-Gauge Flexible Needle: A Multicenter Study. Clinical endoscopy.

[23] Arimura K, Kondo M, Nagashima Y, Kanzaki M, Kobayashi F, Takeyama K (2019). Comparison of tumor cell numbers and 22C3 PD-L1 expression between cryobiopsy and transbronchial biopsy with endobronchial ultrasonography-guide sheath for lung cancer. Respiratory research.

[24] Zarogoulidis P, Huang H, Bai C, Kosmidis C, Trakada G, Veletza L (2017). Endobronchial ultrasound convex probe for lymphoma, sarcoidosis, lung cancer and other thoracic entities. A case series. Respiratory medicine case reports.

